# Biomechanical assessment of balance and posture in subjects with ankylosing spondylitis

**DOI:** 10.1186/1743-0003-9-63

**Published:** 2012-08-29

**Authors:** Zimi Sawacha, Elena Carraro, Silvia Del Din, Annamaria Guiotto, Lara Bonaldo, Leonardo Punzi, Claudio Cobelli, Stefano Masiero

**Affiliations:** 1 Department of Information Engineering, University of Padova, Padova, Italy; 2 Department of Rehabilitation, University of Padova, Padova, Italy; 3 Rheumatology Unit, Department of Clinical and Experimental Medicine, University of Padova, Padova, Italy

**Keywords:** Ankylosing Spondylitis, Postural balance, Biomechanics

## Abstract

**Background:**

Ankylosing spondylitis is a major chronic rheumatic disease that predominantly affects axial joints, determining a rigid spine from the occiput to the sacrum. The dorsal hyperkyphosis may induce the patients to stand in a stooped position with consequent restriction in patients’ daily living activities. The aim of this study was to develop a method for quantitatively and objectively assessing both balance and posture and their mutual relationship in ankylosing spondylitis subjects.

**Methods:**

The data of 12 healthy and 12 ankylosing spondylitis subjects (treated with anti-TNF-α stabilized), with a mean age of 51.42 and 49.42 years; mean BMI of 23.08 and 25.44 kg/m^2^ were collected. Subjects underwent a morphological examination of the spinal mobility by means of a pocket compass needle goniometer, together with an evaluation of both spinal and hip mobility (Bath Ankylosing Spondylitis Metrology Index), and disease activity (Bath Ankylosing Spondylitis Disease Activity Index). Quantitative evaluation of kinematics and balance were performed through a six cameras stereophotogrammetric system and a force plate. Kinematic models together with a test for evaluating balance in different eye level conditions were developed. Head protrusion, trunk flexion-extension, pelvic tilt, hip-knee-ankle flexion-extension were evaluated during Romberg Test, together with centre of pressure parameters.

**Results:**

Each subject was able to accomplish the required task. Subjects’ were comparable for demographic parameters. A significant increment was observed in ankylosing spondylitis subjects for knee joint angle with the target placed at each eye level on both sides (p < 0.042). When considering the pelvic tilt angle a statistically significant reduction was found with the target placed respectively at 10° (p = 0.034) and at 30° (p = 0.019) less than eye level. Furthermore in ankylosing spondylitis subjects both hip (p = 0.048) and ankle (p = 0.029) joints angles differs significantly. When considering the posturographic parameters significant differences were observed for ellipse, center of pressure path and mean velocity (p < 0.04). Goniometric evaluation revealed significant increment of thoracic kyphosis reduction of cervical and lumbar range of motion compared to healthy subjects.

**Conclusions:**

Our findings confirm the need to investigate both balance and posture in ankylosing spondylitis subjects. This methodology could help clinicians to plan rehabilitation treatments.

## Background

Ankylosing spondylitis (AS) is a major chronic rheumatic disease that predominantly affects axial joints, determining a diffuse stiffness and with the advanced stage producing a rigid spine from the occiput to the sacrum, for a chronic process of inflammation of the fibrous connective and bone in the tendon and ligament insertions [1-6]. Prevalence rates of people affected by AS ranges between 0.2% and 1.1% (0.9% in northern European white population) [7,8]. Young population are primarily affected (average age of diagnosis being 24 years), more male than female [7,8].

Sacroiliitis is the distinctive characteristic of AS, with consequent pain in sacral and gluteus region [4]. Also a possible extension of the pain from the thigh to the proximal calf, with alternated course between the two lower limbs have been reported in association with AS [3]. Hence, the clinical manifestations of AS are pain, stiffness, fatigue, reduced spinal mobility and respiratory restriction. Early limitation of spinal mobility has also been identified as one of the most relevant prognostic factors [8]. Other registered symptoms and objective clinical signs of AS are precocious loss of lumbar lordosis, increased dorsal kyphosis and inversion of cervical lordosis, abdominal relaxation for diaphragm breath, hip flexion contracture and consequent knee flexion compensation [2,9].

Some authors reported that the dorsal hyperkyphosis may induce the patients to stand in a stooped position unable to see the horizon [3,5,8]. The main drawback of such condition being the consequent restriction in patients’ daily living activities such as interpersonal communication, driving a car, walking down the street or maintaining personal hygiene [2,4].

Spinal kyphosis in AS subjects has also been associated with a forward and downward shift of the centre of mass (CoM) of the trunk in the sagittal plane, thus inducing a forward and downward shift of the body’s CoM with respect to the base of support [3,5,8]. It has been hypothesized that in AS subjects extension of the hips, flexion of the knees and plantar flexion of the ankles may counterbalance the forward shift of the body CoM relative the base of support [5,8].

However, to date there has been few studies determining the effect of AS on balance. Some authors [5] documented balance alterations on a significant proportion of AS patients both in eyes open and eyes closed conditions, meanwhile Aydog et al. [3] reported AS has no negative effect on postural stability. In their study a clinically significant association was found only between dynamic postural balance and tragus to wall distance. Furthermore the authors have already demonstrated the presence of biomechanics alterations during walking in a group of AS subjects [10]. Statistically significant alterations were observed in the sagittal plane at each joint (P < 0.049), together with hip and knee joint extension moments reduction (P < 0.044) [10].

Loss of balance in AS patients may be associated with severe joint deformities and poor posture. Moreover loss of balance may increase the risk of falls. However in the literature, results concerning assessment of balance in AS subjects are contradictories [3,5,8].

Given this scenario it appears the need of performing an exhaustive evaluation of the consequences of AS also on balance and posture in static conditions. This paper, specifically addressed to the static analysis of the AS subjects’ posture, describes the experimental method developed herein in order to assess the biomechanical features in the interaction between balance and posture. As methodological development it was applied on a limited sample of subjects to verify feasibility and validity before being transferred to a wide population of subjects. Hence, a quantitative multi-factor analysis was applied in static conditions, as previously performed during gait [10]. Moreover the methodology proposed herein enables to assess posture’s modification during balance alterations. This allows identification of distinct postural strategies used by AS subjects in order to cope with poor balance.

This was accomplished by means of clinical and instrumental analysis, which allows quantitative evaluation of kinematic and balance through motion capture systems and force plates. In this context a kinematic model was developed together with a test for evaluating balance in different eye level conditions like what was previously performed in Benedetti et al. [11] while evaluating the effects of a physical activity program on flexed posture elderly individuals.

## Methods

### Subjects

Twelve healthy (control subjects (CS)) and twelve AS subjects have been analyzed, with a mean age respectively of 51.42 (13.91) and 49.42 (10.47) years; a mean BMI respectively of 23.08 (2.37) and 25.44 (3.19) kg/m^2^ (10 female and 14 male). The mean of AS subjects disease duration was 9.64 ± 6.6 years. Inclusion criteria were: all patients with AS met the most recent modified New York criteria [9] and were treated with anti-TNF-α with a stable clinical picture (i.e., with a change in the Bath Ankylosing Spondylitis Disease Activity Index (BASDAI: score between 1 and 10) of no more than ± 1/10 units in the previous 3 months [1].

Exclusion criteria were age older than 70 years, concomitant cardiovascular, neurological or psychiatric disease, and severe visual or auditory impairments (reduced visual acuity was accepted if adequately corrected). Patients with other orthopedic disease or previous surgery at the spine and on the lower extremities were also excluded. All AS subjects were consecutively recruited from the patients attending the outpatient clinic of the Rehabilitation Department of the University of Padova (Italy). CS consisted of normal subjects enrolled among health personnel of the same Rehabilitation Department; all these subjects had no neurological or orthopedic disease and no severe visual or auditory impairments. The study was approved by the hospital’s ethics committee and informed consent was obtained from all patients and controls.

### Clinical assessment

Patients were evaluated [10] by means of the Bath Ankylosing Spondylitis Metrology Index (BASMI) [12], Bath Ankylosing Spondylitis Disease Activity Index (BASDAI) [13] and Bath Ankylosing Spondylitis Functional Index (BASFI) [14].

The manual muscle strength test was performed on the lower limbs using Medical Research Council Score [15].

Subjects underwent a morphological examination of the spine using a specific assessment tools [16]. Both thoracic kyphosis and lumbar lordosis were measured in orthostatic position and the active range of motion (A-ROM) of cervical, thoracic and lumbar spine were evaluated by means of a pocket compass needle goniometer (IncliMed^®^, patent N° 0001331516, University of Padova) [16].

**Figure 1:**
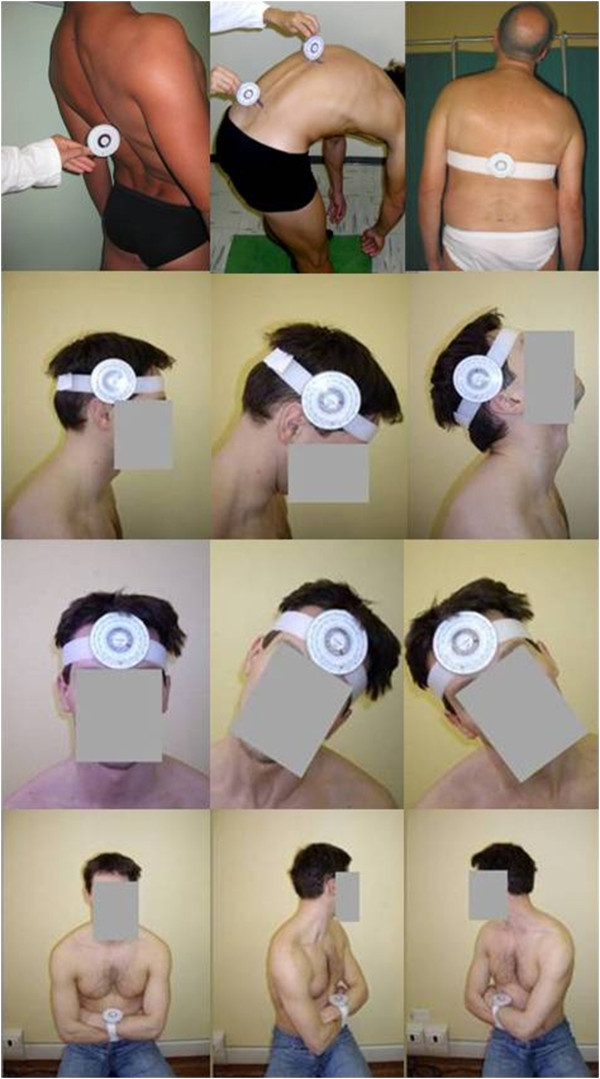
**Subjects while undergoing a morphological examination of the spine using a specific assessment tool.** Both kyphosis and lumbar lordosis were measured and the range of motion of cervical and thoraco-lumbar spine through a pocket compass needle goniometer (IncliMed^®^). From the top to the bottom and from left to right: first the subject is standing barefoot while the goniometer is placed orthogonal to the spine in correspondence of vertebra T12 performs the maximum extension with his knee straighten, and with two goniometers placed in T12 and S2 performs the maximum flexion of thoraco-lumbar spine; the subject stands and the goniometer is positioned flat and parallel to the spine in correspondence of T12 and performs the lateral thoraco-lumbar inclination; the subject seats with the goniometer fixed in neutral position; in this position the subject performs one head flexion and extension; the subject seats with the goniometer fixed in neutral position; the subject performs one head lateral left and right inclination; the subject seats with the goniometer fixed in neutral position; the subject performs left and right thoraco-lumbar rotation.

In this context three angles were measured with the subject standing barefoot while the goniometer was placed orthogonal to the spine in the following three positions (see Figure 1):

T1: under the spinous process of vertebra C7 (α = angle between T1 and vertical axis)

T12: in correspondence of the spinous process of vertebra T12 (β = angle between T12 and vertical axis)

S2: in correspondence of the line connecting the posterior superior iliac spines (γ = angle between S2 and vertical axis).

A measure of the thoracic kyphosis was then obtained by combining the angle between T1 and T12 (α + β), and a measure of the lumbar lordosis by combining the angle between T12 and S2 (β + γ).

In order to evaluate the cervical A-ROM the subject was asked to perform one cervical flexion and extension, right and left rotation and inclination on the right and left side while seating with the goniometer fixed as in Figure 1. The latter was then secured to the arms (see Figure 1) by a Velcro strap when evaluating the thoraco-lumbar rotation. Lumbar flexion-extension was recorded by means of the goniometer positioned orthogonal to the spine in correspondence of T12 and of S2 (following the above described instructions). The subject was then asked to stand with his knee straighten and to perform the maximum flexion and the maximum extension. The trunk flexion comprises the contribution of two components, one lumbar (measured by means of a goniometer positioned in correspondence of T12) and one sacral (measured by means of a goniometer positioned in correspondence of S2), hence the lumbar flexion-extension angle was obtained as the difference between the angles measured respectively at T12 and S2 in these positions. The lateral thoraco-lumbar inclination was evaluated with the subject standing and positioning the goniometer flat and parallel to the spine in correspondence of T12 fixed by a Velcro [16].

### Instrumental assessment

**Figure 2:**
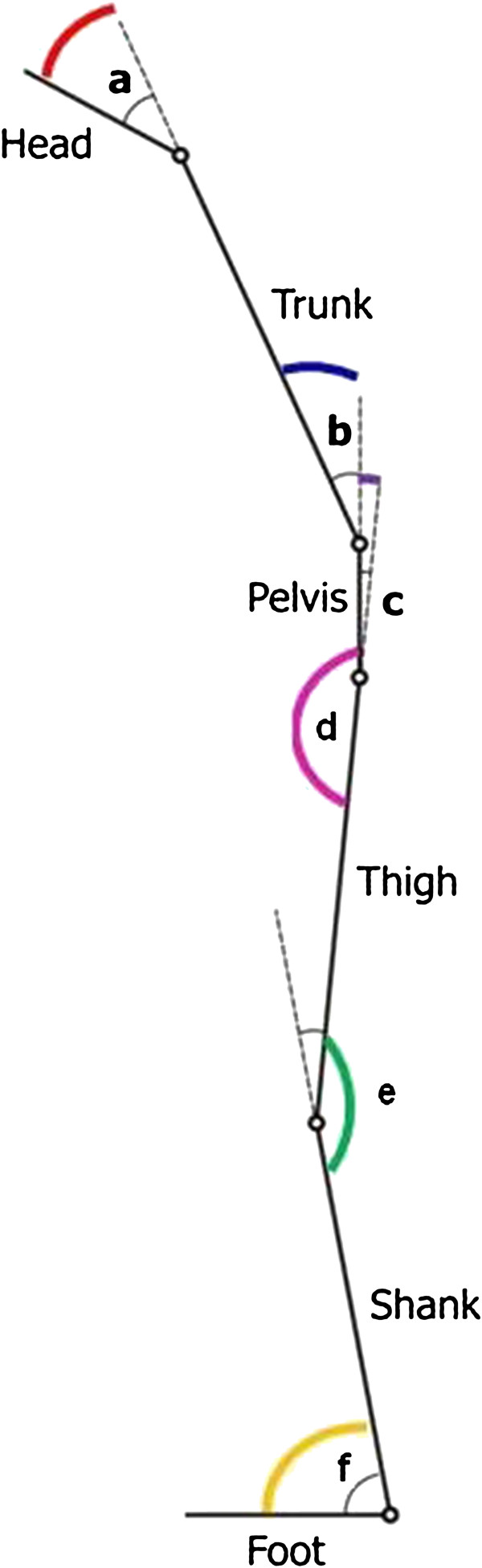
**Description of the joint angles in the sagittal plane.** Head protrusion (**a**), trunk flexion-extension (**b**), pelvic tilt (**c**), hip flexion-extension (**d**), knee flexion-extension (**e**), ankle flexion-extension (f).

Posture was evaluated by means of a six cameras stereophotogrammetric system (60-120 Hz, BTS S.r.l., Padova, Italy) synchronized with a Bertec force plate (FP4060-10, 960 Hz). A modified version of Leardini et al. 2007 [10,11,17,18] was adopted for the kinematic analysis based on the Cast Protocol [19] which entails the application of 3 extra reflective markers on the head in correspondence of glabella, right and left temporomandibular joints. The following joint angles in the sagittal plane were evaluated as [11,17,18].

head protrusion (HP): the supplementary angle to the angle between head and upper trunk upper trunk flexion-extension (UTFE): the angle between upper trunk (defined by means of the 7^th^ cervical vertebra and the right and left acromions) and trunk (see Figure 2)

trunk flexion-extension (TFE): the angle between trunk (defined by means of the 5^th^ lumbar vertebra and the right and left acromions) and pelvis (see Figure 2)

pelvic tilt (PT): is the angle of rotation about the medio-lateral axis of the pelvis

right and left hip flexion-extension (rHFE, lHFE): the angle between pelvis and femur

right and left knee flexion-extension (rKFE, lKFE): the angle between femur and shank

right and left ankle flexion-extension (rAFE, lAFE): the angle between shank and foot

Subjects were asked to stand for 60 seconds in an upright position over the force plate, with the feet 30° apart and their arms along the body and to look at a small achromatic circular target placed about 1 meter from the eyes [18,20], at 7 different heights: eye height, ±10°, ±20°, ±30° than their eye level (e.l.). Romberg test was also performed entailing performing two static acquisitions (stand for 60 seconds in an upright position over the force plate, with the feet 30° apart and their arms along the body), one in eyes open (e.o.) and one in eyes closed (e.c.) conditions. Both kinematic and centre of ground reaction forces data were collected.

The total body center of pressure (CoP) trajectory over the support surface was computed from the vertical force of the force platform, which were recorded for 60 seconds at 960 Hz and then filtered by a 3rd order, low-pass Butterworth filter (cut-off frequency 5 Hz). The first 20 seconds of the acquisition were excluded from the analysis [18,21,22].

A total of 9 CoP measures were computed from the CoP displacement in the horizontal plane [21-24]. In the time domain, we obtained 3 measures that characterized the CoP trajectory over the support surface, 2 measures that estimated the area covered by the CoP, and 3 measures that estimated the velocity of body sway over the support surface. The following CoP-based measures were computed [21,22]: Sway area (area included in CoP displacement per unit of time (mm^2^/s)), Ellipse 95%, the CoP coordinate time series, the CoP coordinate time series in antero-posterior (AP) and medio-lateral (ML) directions, mean velocity (total CoP trajectory length/trial duration (mm/s)), mean velocity in AP and ML directions.

### Statistical analysis

Continuous data were summarized in terms of means and standard deviation of the mean. The differences between AS and CS were investigated by means of the unpaired *T*-Test followed by a Tukey-Kramer test (Matlab software function Ttest) when the variances were homogenous (verified with Lilliefors test, Matlab software function lillietest) and the Kruskal*-*Wallis test (Matlab software function kruskalwallis) followed by a Tukey-Kramer test when the variances were not homogeneous; p-values under 0.05 were considered to be significant. Subjects who averaged more than 3.5 standard deviations from the mean were classified as outliers and removed from the statistical analysis [25].

The evaluation of the confidence intervals for the observed proportions was performed with the staRt Package of R statistical software [26].

## Results

**Figure 3:**
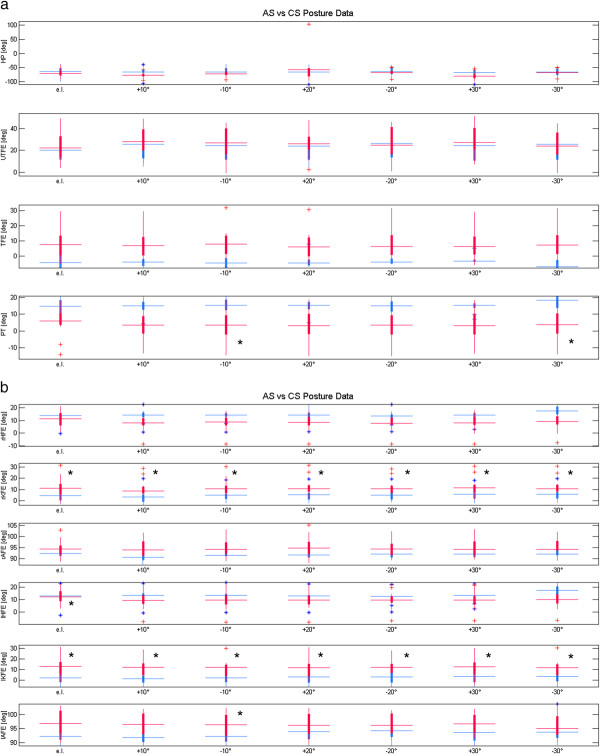
**Boxplots of the kinematic parameters: Ankylosing spondylitis (AS) in red, Control Subjects (CS) in blue.** Horizontal axis depicts the 7 eyes eights (eyes level = e.l.); from top to bottom vertical axes represent: the head protrusion (HP), the upper trunk flexion-extension (UTFE), the trunk flexion-extension (TFE), the pelvic tilt (PT) (Figure 3a), the right hip flexion-extension (rHFE), the right knee flexion-extension (rKFE), the right ankle flexion-extension (rAFE), the left hip flexion-extension (lHFE), the left knee flexion-extension (lKFE), and the left ankle flexion-extension (lAFE) (Figure 3b). * Statistical significance (*P* < 0.05).

**Figure 4:**
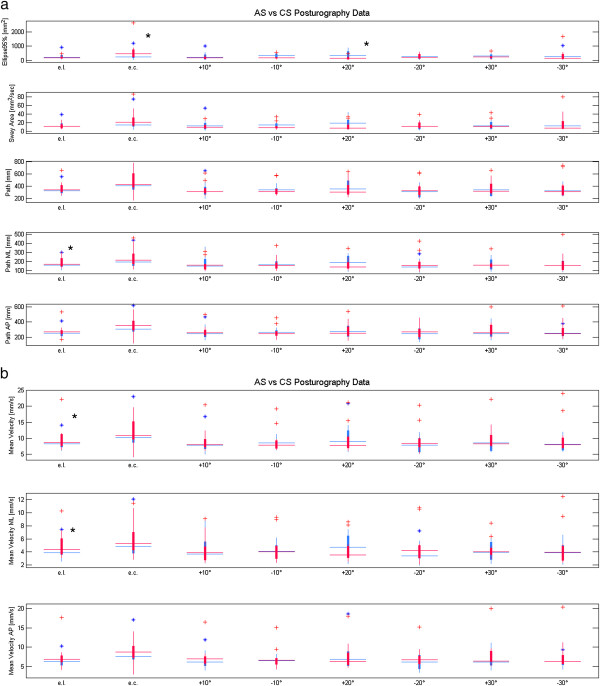
**Boxplots of the posturographic parameters: Ankylosing spondylitis (AS) in red Control Subjects (CS) in blue.** Horizontal axis depicts the 7 eyes eights (eyes level = e.l., eyes closed = e.c.); from top to bottom vertical axes represent: the ellipse 95% (Ellipse95%), the sway area (Sway Area), the total path (Path), the path in medio-lateral direction (Path ML), the path in anterior-posterior direction (Path AP) (Figure 4a), the total mean velocity (Mean Velocity), the mean velocity in medio-lateral direction (Mean Velocity ML), and the mean velocity in anterior-posterior direction (Mean Velocity AP) (Figure 4b). * Statistical significance (* P* < 0.05).

Each subject was able to accomplish the required task. Subjects’ demographic and clinical characteristics were reported in Table 1. The mean BASMI, BASDAI and BASFI scores were respectively 3.38 (1.26), 2.50 (1.59), 2.22 (1.32). The manual muscle test showed normal of strength. Goniometric evaluation revealed in AS subjects a significant increment of thoracic kyphosis but no lumbar lordosis, and a significant reduction of both cervical and lumbar A-ROM was registered compared to healthy subjects. A box plot representation of the results obtained from both joint angles and posturographic parameters has been reported in Figures 3 and 4. In this contest the data relative to subjects identified as outliers were also reported. Statistically significant differences were observed for knee joint angle with the target placed at each e.l. on both sides (p < 0.042). When considering the pelvic tilt angle statistically significant differences were found with the target placed respectively at 10° (p = 0.034) and at 30° (p = 0.019) less than e.l. Furthermore the left side registered significant differences in correspondence of hip (at e.l. p = 0.048) and ankle (at 10° less than e.l., p = 0.029) joints.

**Table 1:** Subjects demographic and clinical characteristics (mean and standard deviation)

	**CS**	**AS**	**P**
**Age [years]**	**51.4 (13.9)**	**49.4 (10.5)**	**0.694**
**Sex (male/female)**	**6/6**	**8/4**	**0.430**
**Disease duration [years]**		**9.6 (6.6)**	
**BMI [kg/m**^**2**^**]**	**23.1 (2.4)**	**25.4 (3.2)**	**0.052**
**Kyphosis**	**45.3 (9.4)**	**54.8 (12.2)**	0.044*
**Lordosis**	**36.2 (11.2)**	**28.8 (14.7)**	**0.182**
**Pelvic anteversion**	**5.7 (2.3)**	**1.00 (3.6)**	0.016*
**Flexion-extension of lumbar spine**	**69 (14.8)**	**31.3 (13.8)**	0.001*
**Lateral inclination of thoraco-lumbar spine**	**58.5 (13.3)**	**29.1 (18.2)**	0.001*
**Rotation of thoraco-lumbar spine**	**90.3 (12.8)**	**70.2 (23.9)**	0.016*
**Total range of motion of thoraco-lumbar spine**	**221.5 (30.1)**	**130.7 (44.7)**	0.001*
**Flexion-extension of cervical spine**	**121.2 (15.0)**	**90.5 (32.8)**	0.007*
**Lateral inclination of cervical spine**	**78.4 (10.5)**	**52.8 (23.7)**	0.002*
**Rotation of cervical spine**	**118.3 (15.5)**	**102.08 (39.0)**	**0.193**
**Total range of motion of cervical spine**	**318.0 (25.0)**	**245.4 (84.7)**	0.009*

* Statistical significance (*P* < 0.05).

When considering the posturographic parameters results on healthy subjects were found consistent with previous literature [21]. Significant differences were observed, between AS and CS for the following variables: ellipse in e.c. condition (p = 0.03), CoP path in ML direction in e.o. condition (p = 0.007), CoP mean velocity in e.o. condition (p = 0.04), CoP mean velocity in ML direction in e.o. condition (p = 0.02), ellipse (p = 0.02) with the target placed at +20° than their eye level. Nevertheless the mean velocity AP registered a p value of 0.05 when the target was placed at 30° less than e.l.

## Discussion

The key finding of the present paper is that instrumental assessment of both balance and posture allowed us to measure either the global postural alignment or spine mobility and its influence on AS balance. Only clinical and non invasive instrumental evaluation of the spine were performed on each subject within the present study. This was chosen in agreement with Assessment in Spondyloarthritis International Society (ASAS), whose guidelines suggests that disease monitoring of patients with AS should include: patient history (for example, questionnaires), clinical parameters, laboratory tests, and imaging. The frequency of monitoring should be decided on an individual basis depending on symptoms, severity, and drug treatment [1]. In patients with AS in TNF alpha treatment the imaging assessments should be performed at baseline, but more rarely in the follow up.

The experimental set up can be considered simple and the study was carried out with a good compliance by the patients. Nowadays three dimensional motion analysis is highly diffuse in clinical settings [23], and allows quantitative and objective evaluation of biomechanics parameters while subjects are performing specific tasks. In the present study it was successfully applied to quantitatively evaluate both AS subjects’ balance and posture during standing.

AS subjects exhibited a poor posture with reduced pelvic anteversion and excessive trunk and knee flexion displayed at each task, even though only the last one resulted statistically significant. These postural modifications could be associated with a forward and downward shift of the CoM of the trunk in the sagittal plane, which has been previously assessed in relation to an excessive thoracic kyphosis [3]. In particular, excessive knee flexion has been described by other authors as a typical characteristic of AS posture which, as a major consequence, produces earlier fatigue than standing with extended legs due to considerable effort of quadriceps muscle [27]. The present method was also able to highlight a changing in AS posture strategy when target was placed at 10° less than e.l. In this condition AS subjects’ ankle dorsiflexion increased while both their pelvic anteversion and hip flexion were reduced. Furthermore a reduction in pelvic antiversion was observed also at 30° less than e.l. This could determine hip joint extensor muscle contractures. However this extension of the hips and a posterior rotation of the pelvis could serve as a strategy in order to improve their field of vision, as was previously hypothesized by Bot et al. [8]. The same can be assessed for the reported increment in ankle and knee flexion that represents a compensatory mechanism to the displacement of the trunk CoM. Indeed also Bot et al. [8] reported similar results for three out of four AS patients, when asked to adopt a posture, which enables them to see the horizon. With regard to relationships between postural instability and spinal curvature this has been assessed for osteoporosis-related kyphosis subjects [28]. In these patients were also registered excessive thoracic kyphosis and presence of compensatory modifications of the lumbar lordosis [29]. Even though the study was carried out on a different population, it has been reported that patients with thoracic hyperkyphosis displayed greater use of hip or ankle joint to maintain balance. This was justified with the significantly lower both hip abductor and weight-bearing muscles strength registered in osteoporosis-related kyphosis subjects [28-31]. A similar condition can be hypothesized also in AS subjects where reduced capacity of shock absorption has been also documented, which could be associated with weakness of weight-bearing muscles [10,32]. An overall analysis of results of the postural analysis revealed the presence of a strategy in order to maintain their balance while performing different tasks in AS subjects. So far these subjects answer to an increment of their knee flexion with a dorsiflexed ankle. They answer to an increment of the head protrusion with an increment of the trunk flexion and a reduction of the upper trunk flexion angle. Since AS pathology affects axial joints the above described strategy was accomplished bilaterally. The angles chosen in the postural instrumental assessment can be considered clinical meaningful; indeed they can be referred to precise compensatory axial deviations to kyphosis. This has been previously reported also by Benedetti et al. [11] in the assessment of elderly subjects posture. Based on these results, we can suggest that in a rehabilitative program for AS subjects it could be useful introducing exercises for strengthening weight- bearing muscles: strengthening the back extensor could counterbalance the attitude to flexed posture, meanwhile strengthening and stretching the muscle of the kinetics posterior muscular chain (as hamstrings and triceps muscles) could either reduce the fatigue or increase the endurance. This finds agreement in Masiero et al. 2011 [16].

While considering the proposed instrumental balance evaluation test it included postural assessment during different conditions. This was chosen in order to evaluate the ability of AS subject to cope with a posture, which prevents them from seeing the horizon. As expected AS people who are characterized by a stoop posture when asked to watch a target at a higher or lower level than eyes eight adopted compensation strategies. In particular they increased their knee flexion with a dorsiflexed ankle. It should be noticed that this specific methodology allows identifying the presence of balance impairment in AS subjects. Indeed an increment of both Ellipse and CoP path was observed on each task with respect to the CS group, their mean values representing twice the one of CS group. Furthermore the values of these parameters increased drastically for the task of ±20° and ±30° than e.l. These preliminary findings seem to suggest the presence of balance impairment in correspondence of an increment of the target angle in both directions. This could be associated with their characteristic stooped posture together with excessive kyphosis, which by changing their trunk CoM position could affect their balance. Furthermore apparently in contrast with the literature [8] AS subjects displayed balance impairment also during Romberg test both in e.c. and e.o. condition. These results suggest that AS subjects balance problems should be taken into account when planning a rehabilitative treatment. In this context proprioceptive exercise that increases both the static and dynamic postural stability should be included.

When considering the clinical characteristics of the studied subjects it should be taken into account that AS and CS subjects were comparable for demographic parameters. BASMI, BASDAI and BASFI scores of our patients were found to be lower than the patient scores in other studies (at variance with previous literature our patients were treated with anti-TNF-α) [30,31,33-35]. In order to allow clinicians to take advantage of this methodology the present paper reported also the clinical postural assessment as reported in the literature [8,9,11,33]. When comparing the multidimensional goniometric clinical assessment a statistically significant increment was noticed in AS kyphosis angle (p = 0.044), AS pelvic retroversion (p = 0.01) together with a reduction in the A-ROM of cervical and lumbar spine on each plane (p < 0.016) with the exclusion of the cervical rotation. These results were found in agreement with the consequences of AS reported in the literature which affecting the axial joints, determines a diffuse stiffness and produces a rigid spine from the occiput to the sacrum [3]. Finally it can be hypothesized that these results could be related to the Pelvis–shoulder coordination alterations found by Mangone et al. in AS subjects [36], even though their findings were related to walking conditions.

## Conclusions

A method to assess both balance and posture in AS subjects was developed. The former combines clinical and instrumental analysis in order to obtain quantitative evaluation of kinematic and balance through motion capture systems and force plates. In this context a kinematic model was developed together with a test for evaluating balance in different eye level conditions. AS subjects exhibited significant increment in their kyphosis angle, pelvic retroversion and a reduction in the A-ROM of cervical and lumbar spine. A significant increment was observed in AS knee joint angle together with a reduction of pelvic tilt angle. Finally the CoP path increased significantly during Romberg Test in eyes closed condition (p = 0.04). The methodology allowed identification of the relationship between kinematic and balance alterations during static posture. Results could be considered very useful as baseline for clinician who should plan exercise protocols or rehabilitation treatments [16,29,30,34,35,37]. Of course further studies including a larger sample of subjects should be performed in order to confirm our results.

## Competing interests

Each of the authors has read and concurs with the content in the final manuscript. The contributing authors guarantee that this manuscript has not been submitted, nor published elsewhere. Each of the authors declares that don’t have any financial and non-financial competing interests.

## Authors' contributions

Each of the authors has read and concurs with the content in the final manuscript. ZS, EC, SM and CC participated in conceiving the study. ZS, EC, SM and CC participated in its design and coordination and carried out the drafting of the manuscript. SD helped to draft the manuscript. ZS, AG, SD carried out the experimental part of the study relatives to the motion analysis data collection and carried out and coordinated the data analysis. AG, SD participated to the experimental part of the study relatives to the motion analysis data collection and performed the data analysis. EC, LB carried out the experimental part of the study relatives to the clinical evaluation and participated to the motion analysis data collection. LP made the diagnosis of AS, followed the pharmacological treatment and supervised the manuscript. All authors read and approved the final manuscript.
